# Supplementary figures and images for: Rpl24Bst mutation suppresses colorectal cancer by promoting eEF2 phosphorylation via eEF2K
Source: eLife. 2021 Dec 13;10:e69729. doi: 10.7554/eLife.69729 (PMC8668188; doi:10.7554/eLife.69729)

Figure 1F

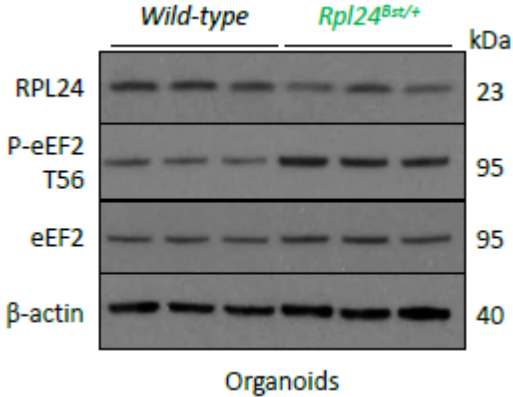

RPL24

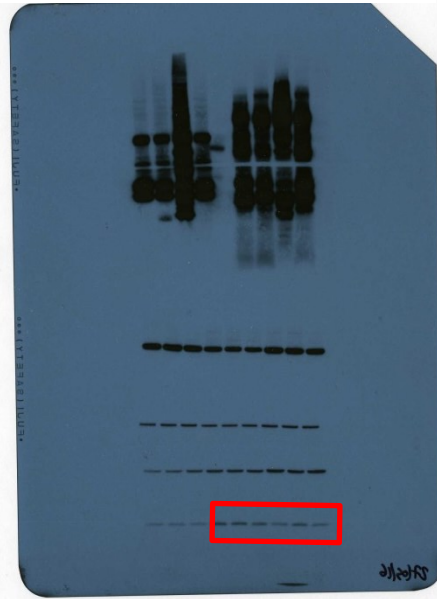

eEF2 T56-P

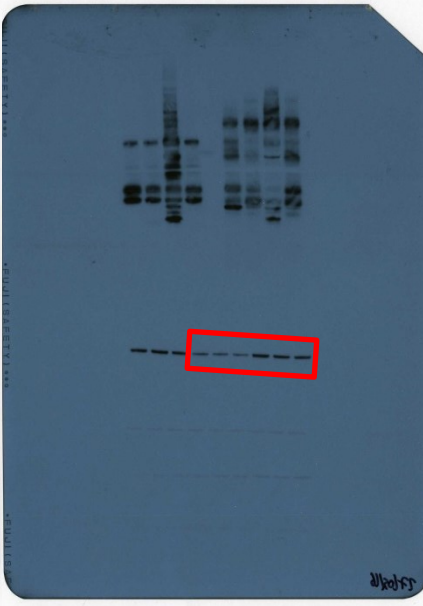

eEF2

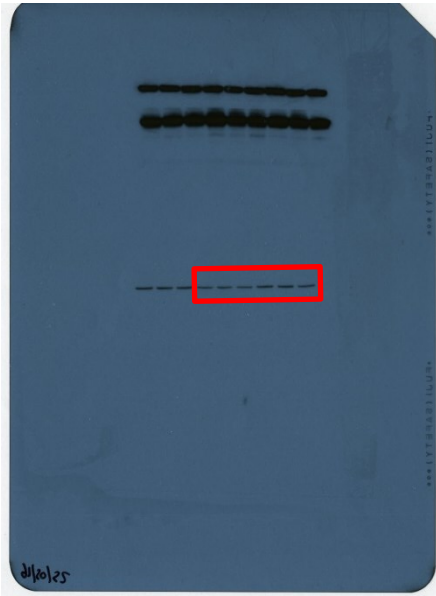

β-actin

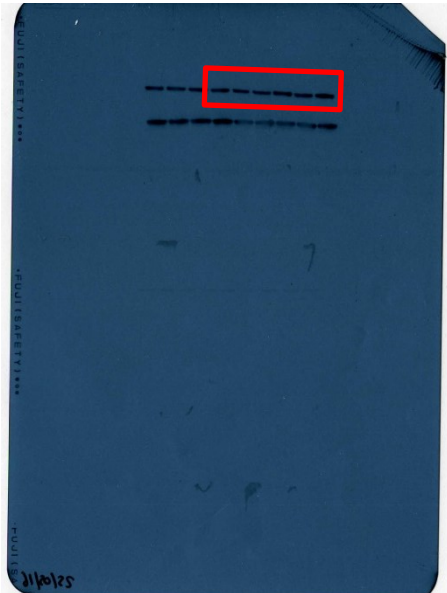

Supplement: Figure 1—source data 1. — Bottom: original western blotting images with areas for each antibody annotated and boxed in red. [file elife-69729-fig1-data1.pdf]

Figure 3 – figure supplement 2 – source data 1

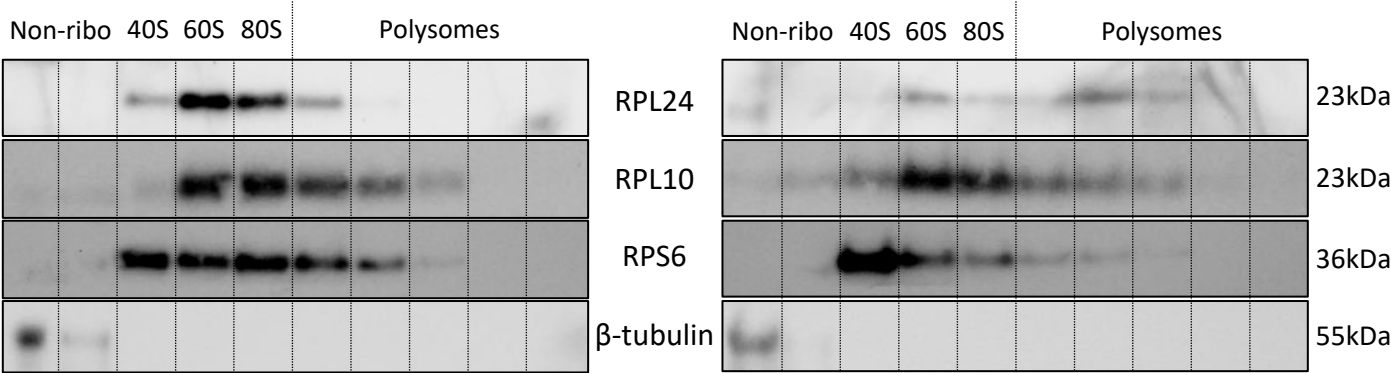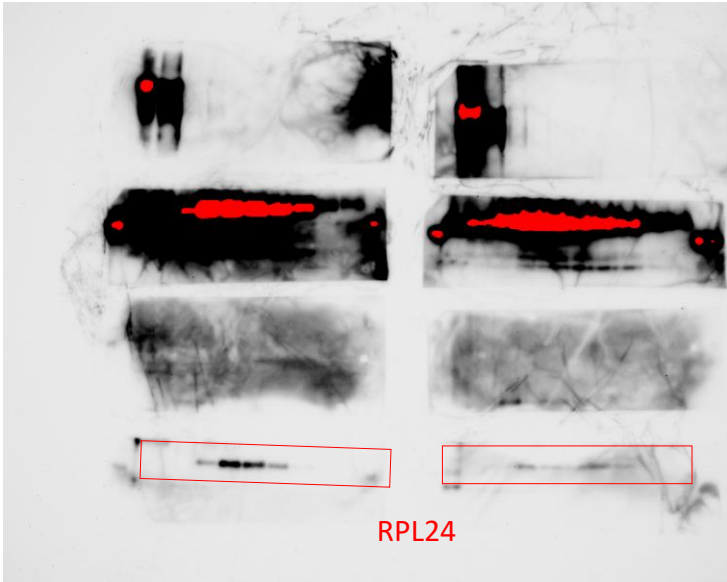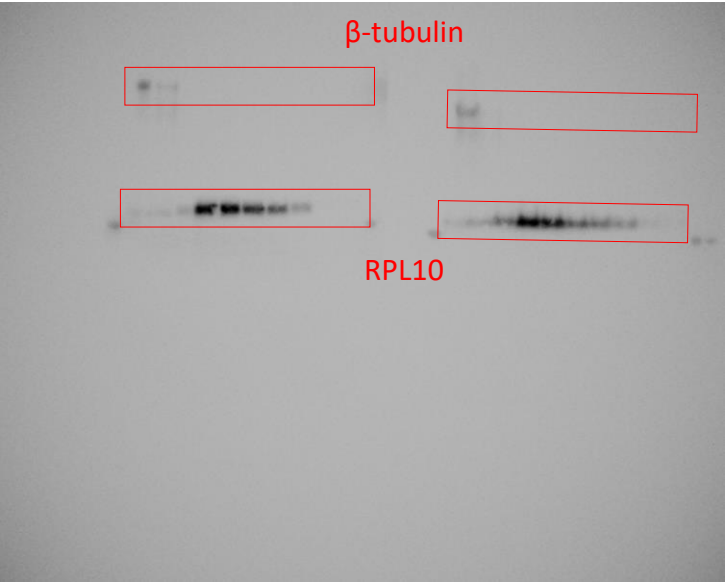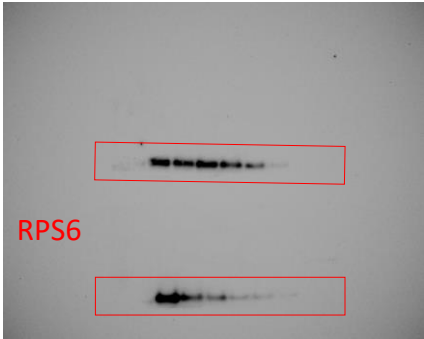

Supplement: Figure 3—figure supplement 2—source data 1. — Bottom: original western blotting images with areas for each antibody annotated and boxed in red. [file elife-69729-fig3-figsupp2-data1.pdf]

Figure 6 – figure supplement 2 – source data 1

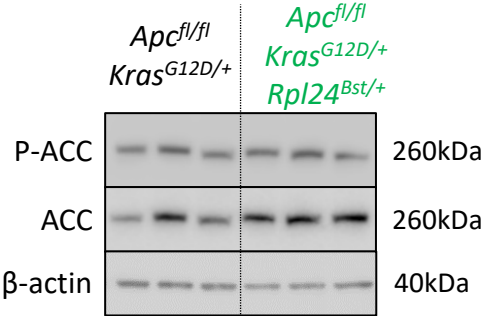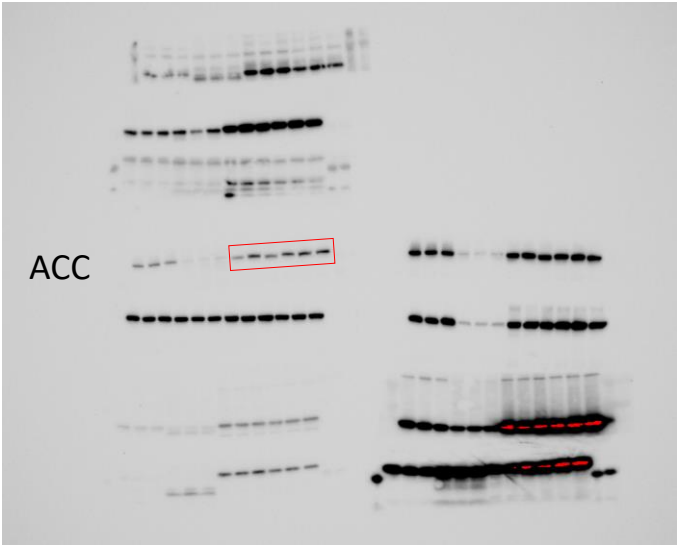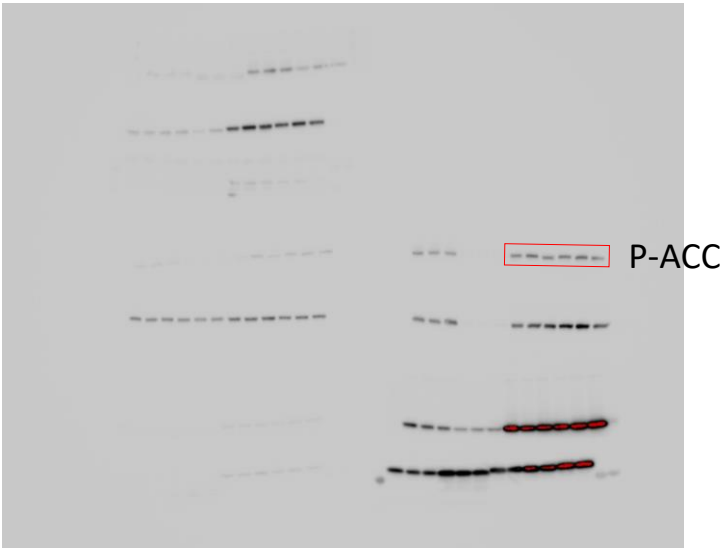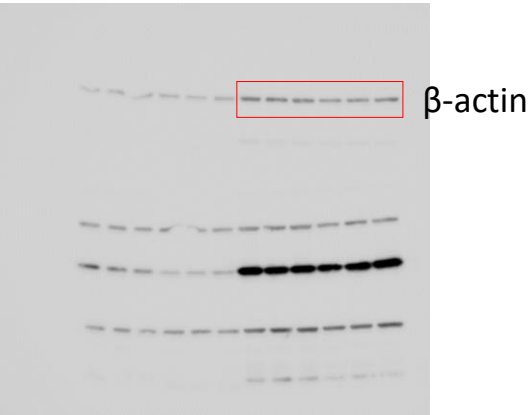

Supplement: Figure 6—figure supplement 2—source data 1. — Right: original western blotting images with areas for each antibody annotated and boxed in red. [file elife-69729-fig6-figsupp2-data1.pdf]
